# Supplementary material for: HCF-2 inhibits cell proliferation and activates differentiation-gene expression programs
Source: Nucleic Acids Res. 2019 May 3;47(11):5792–808. doi: 10.1093/nar/gkz307 (PMC6582346; doi:10.1093/nar/gkz307)
Supplement: gkz307_Supplemental_Files [file gkz307_supplemental_files.zip › Gudkova et al. Supplementary .pdf]

HCF-2 inhibits cell proliferation and activates differentiation-gene expression programs

Daria Gudkova[1], Oleksandr Dergai[1], Viviane Praz[1,2], and Winship Herr[1*]

SUPPLEMENTARY DATA

Supplementary Figures

Figure S1. Related to Figure 1

Figure S2. Related to Figure 3

Figure S3. Related to Figure 3

Figure S4. Related to Figure 4

Figure S5. Related to Figure 5

Figure S6. Related to Figure 6

Figure S8. Related to Figure 7 and Figure S7

Figure S9. Related to Figure 8

Figure S10. Related to Figure 9 and Discussion

Figure S11. Related to Discussion

Supplementary Tables (Separate files)

Table S1. Related to Figure S1 and Figure S4

Table S2. Related to Figure 7, Figure S8

Table S3. Related to Figure 7, Figure 8

Table S4. Related to Figure 8, Figure S9

SUPPLEMENTARY FIGURES

Figure S1. ***(A)*** Alignment of human, rabbit, mouse, and rat HCF-2 protein sequences. The absence of amino acid sequences (488-555, in human HCF-2), encoding by exon 11 is rodent specific. The boxed sequence, representing the region of biggest sequence difference, depicts the mHCF-2 antigen used for rabbit immunization. ***(B, C)*** Coomassie-stained PAGE of immunoprecipitated (IP) hHCF-2 from HEK-293 cells (***B***) and mHCF-2 from MEF (***C***). Arrows identify the hHCF-2 and mHCF-2 bands of different size. IgG, non-immune serum. Asterisk, antibody heavy chain. ***(D)*** Results of MS of indicated bands in **B** and **C**. ***(E)*** Immunoblot of endogenous mHCF-2 from indicated mouse cell extracts (lanes 1-4) with affinity purified α-HCF-2 antibody. ***(F)*** Immunoblot of human and mouse HCF-2. Lane 1, Cherry-hHCF-2 fusion protein; lane 2, human HEK-293 cell extract; lanes 3 and 4, mouse C2C12 and MEF cell extracts, respectively. Tubulin, loading control.

Figure S2. ***(A)*** Immunostaining of MEF cells either with pre-immune serum (left bottom) or α-HCF-2 antibody (right bottom, green). Upper panels, nuclear DAPI staining (blue). ***(B)*** HEK-293 cells were treated with siRNA against luciferase (left) or HCF-2 (right) for 72 h and then stained with α-HCF-2 antibody (green, lower panels). The upper panels show cell nuclei stained with DAPI (blue). ***(C)*** HEK-293 cells were treated for 4 h with (from left to right) DMSO (vehicle), 50 ng/ml and 5 ug/ml actinomycin D (ActD), 1 uM BMH-21, and 25 uM etoposide. Cells were stained with α-NPM antibody (red, upper panels) for nucleolar integrity and α-HCF-2 (green, lower panels). Dashed line denotes the nuclear area.

Figure S3. Analysis of a small-scale chromatin and nucleolar fractionation procedures. (***A***) Schematics of chromatin (upper) and nucleolar (lower) fractionation procedures. (***B***) Chromatin fractionation of HEK-293 cells showing S2 (lane 1), S3 (lane 2), and P3 (lane 3) fractions and whole cell lysate (lane 4) stained with α-MEK and α-tubulin antibodies for cytosolic proteins; α-HCF-1 and α-histone 3 (H3) antibodies for chromatin-bound proteins; and α-RPA194, α-NPM and α-NCL antibodies for nucleolar proteins. (***C***) Nucleolar fractionation of HEK-293 cells showing whole cell lysate (lane 1); cytoplasmic and nuclear (lane 2), and nucleolar (lane 3) fractions. Protein markers are as in ***B***, except for sc-35 for the nucleoplasm.

Figure S4. Immunoblot of NPM in protein complexes immunoprecipitated from MEF extracts with α-HCF-2 antibody. Lane 1, IP with α-HCF-2 antibody; lane 2, IP with negative control IgG; lane 3, whole cell lysate.

Figure S5. ***(A)*** Co-immunostaining of HEK-293 cells expressing either F-Cherry (upper row) or F-Cherry-HCF-2_{WT} (middle and bottom rows) 24 h after induction with doxycycline with either α-HCF-2 or α-NPM antibodies (green). Left panels, nuclear staining with DAPI (blue), right panels, a signal from Cherry. ***(B)*** Alignment of amino acid sequences of Fn3n and Fn3c domains of hHCF-2 (upper line) with hHCF-1 (bottom line). The middle line represents identities (letter) and similarities (+) between the two sequences. The Fn3-1 sequences are boxed in light green and Fn3-2 sequences are boxed in dark green. The Fn3 repeats are linked by 6 amino acid loop (boxed in pink). ***(C)*** PyMOL generated overlay of HCF-2 (green) Fn3n (left) and Fn3c (right) elements with HCF-1 (pink) Fn3nc structure (21).

Figure S6. ***(A)*** MTT assay of HEK-293 cells with either F-Cherry only or F-Cherry-HCF-2_{WT} during 7 days after addition of doxycycline. ***(B)*** F-Cherry-HCF-2_{WT} cells display a number of mitotic defects 72 h after doxycycline induction. Multinucleated cells (white arrows) and micronuclei (yellow arrows) are indicated.

Figure S7. ***(A)*** Normalized read counts mapped to (i) HCF-2-coding sequences (left) resulting from both endogenous and recombinant *HCFC2* sequences and (ii) *HCFC2* 3'UTR sequences (right) from endogenous *HCFC2* sequences only. **(B)** Plot of the two major PCA components of individual RNA-seq samples, dots' labels indicate day after doxycycline addition.

Figure S8. ***(A)*** MA plots of read counts for genes in samples with F-Cherry-HCF-2_{WT} (upper row) and F-Cherry-HCF-2_{Fn3nc*} (lower row) of indicated day versus day 1. Mean of two replicates +1 pseudocount is shown. Genes with absolute value of log2FoldChange >10 were automatically rescaled to 10 are encircled with red. ***(B)*** Venn diagrams represent common and unique genes found differentially expressed in both F-Cherry-HCF-2_{WT} (blue) and F-Cherry-HCF-2_{Fn3nc*} (green) samples at day 6 with respect to day 1.

Figure S9. Selected GSEA enrichment plots for Hallmark gene sets with high absolute value of normalized enrichment scores (NES) based on Gene Set Enrichment Analysis of differentially expressed genes of day 1 and day 6 of the HCF-2_{WT}-samples. Only the hallmarks most relevant to observed phenotype are shown (for full list of GSEA Hallmark sets see Supplementary Table S4). ***(A)*** Down-regulated and ***(B)*** up-regulated GSEA Hallmark sets. ***(C)*** Up-regulated Hallmark sets with fewer than 50 genes. Numbers in the right upper corner indicate ratio of genes found differentially expressed to total number of genes in hallmark gene set.

Figure S10. Comparison of a human HCF-1_{PRO}-repeat sequence with two HCF-1_{PRO} repeat-like sequences in Chimaera HCF-1-like (1007-1031 and 1045-1070 aa) and HCF-2-like (920-945 and 959-984 aa) proteins. Cleavage site and Threonine-rich sequences are shown in red and blue respectively. Dot represents identity with human HCF-1_{PRO} repeat 1 sequence.

Figure S11. Analysis of public data sets for *HCFC2* expression (RPKM or counts) in fish ***(A)***, frog ***(B)*** and mouse ***(C)*** early embryonic stages (61-63). Respective developmental stages for each species are shown. Stages, corresponding to the formation of the blastula and the gastrula are boxed in pink and blue respectively.

SUPPLEMENTARY TABLES

Table S1. List of proteins identified by mass spectrometry of immunoprecipitated mHCF-2 complexes from MEF extracts. Ctrl, non-immune rabbit IgG

Table S2. List of genes detected in RNA-seq of HEK-293 cells with induced HCF-2_{WT} or HCF-2_{Fn3nc*} synthesis. Table S2 shows list of genes with FPKM > 1 which were kept for differential expression analysis done with DESeq2. Columns J and Z (entitled as “Average of expression") show averaged counts for samples with induced expression of HCF-2_{WT} and HCF-2_{Fn3nc*} respectively. Cluster identifiers produced by

PAM (see Materials and Methods) are shown in column I and NA means that gene was not attributed to any of clusters, e.g. gene was stably expressed across all samples. lfcSE is standard error of log2Foldchange estimate; stat is t-statistic returned by Wald test from DESeq2 package. Genes' genomic positions are shown in respect to GRCh37 (Hg19) genome version, gene annotations were obtained from www.gencodegenes.org.

Table S3. List of GO terms associated with genes in PAM clusters I–IV. GO terms with FDR < 0.05 are listed.

Table S4. GSEA report for differentially expressed gene sets in F-Cherry-HCF-2_{WT} and F-Cherry-HCF-2_{Fn3nc*} samples. Results of GSEA run on 50 hallmark pathways for sets of differentially expressed genes for HCF-2_{WT} or HCF-2_{Fn3nc*} between day 6 and day 1. ES is enrichment score, NES - normalized enrichment score. ORIGINAL SIZE column represents number of genes annotated to hallmark pathway, SIZE column gives number of differentially expressed genes per pathway. For details visit https://software.broadinstitute.org/gsea/doc/GSEAUserGuideFrame.html

Figure S1

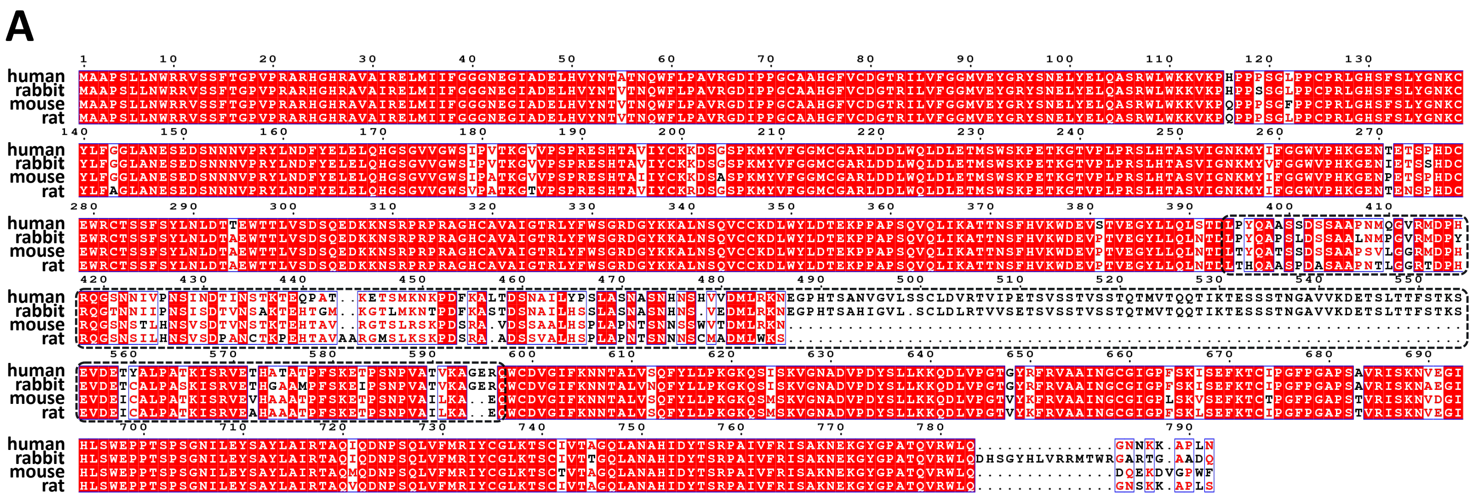

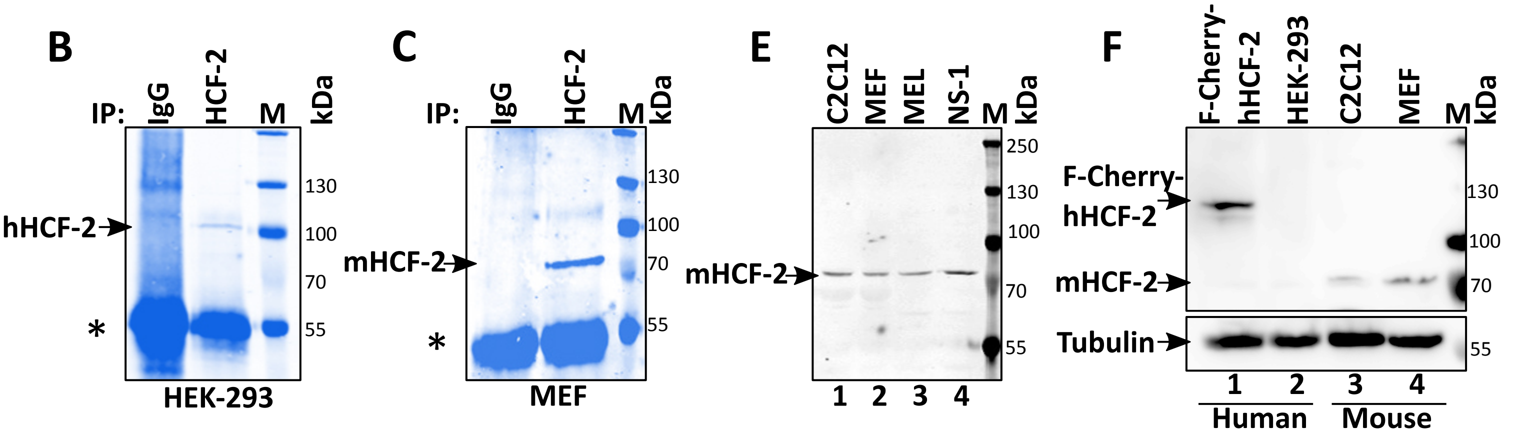

D

IP from HEK-293:

	Identified proteins	Gene symbol	Molecular weight	Spectral counts vs IgG
1	Host cell factor 2	HCFC2_HUMAN	87 kDa	121
2	BTB/POZ domain containing KCTD3	KCTD3_HUMAN	89 kDa	103
3	Nucleolar RNA helicase 2	DDX21_HUMAN	87 kDa	100
4	Centromere protein F	CENPF_HUMAN	368 kDa	89
5	RNA helicase DHX15	DHX15_HUMAN	91 kDa	80

IP from MEF:

	Identified proteins	Gene symbol	Molecular weight	Spectral counts vs IgG
1	Host cell factor 2	G5E837_MOUSE	79 kDa	59
2	78 kDa glucose-regulated protein	GRP78_MOUSE	72 kDa	17
3	Prelamin-A	LMNA_MOUSE	74 kDa	12
4	RNA-binding protein 14	RBM14_MOUSE	69 kDa	6
5	Stress-70 protein, mitochondrial	GRP75_MOUSE	73 kDa	6

Figure S2

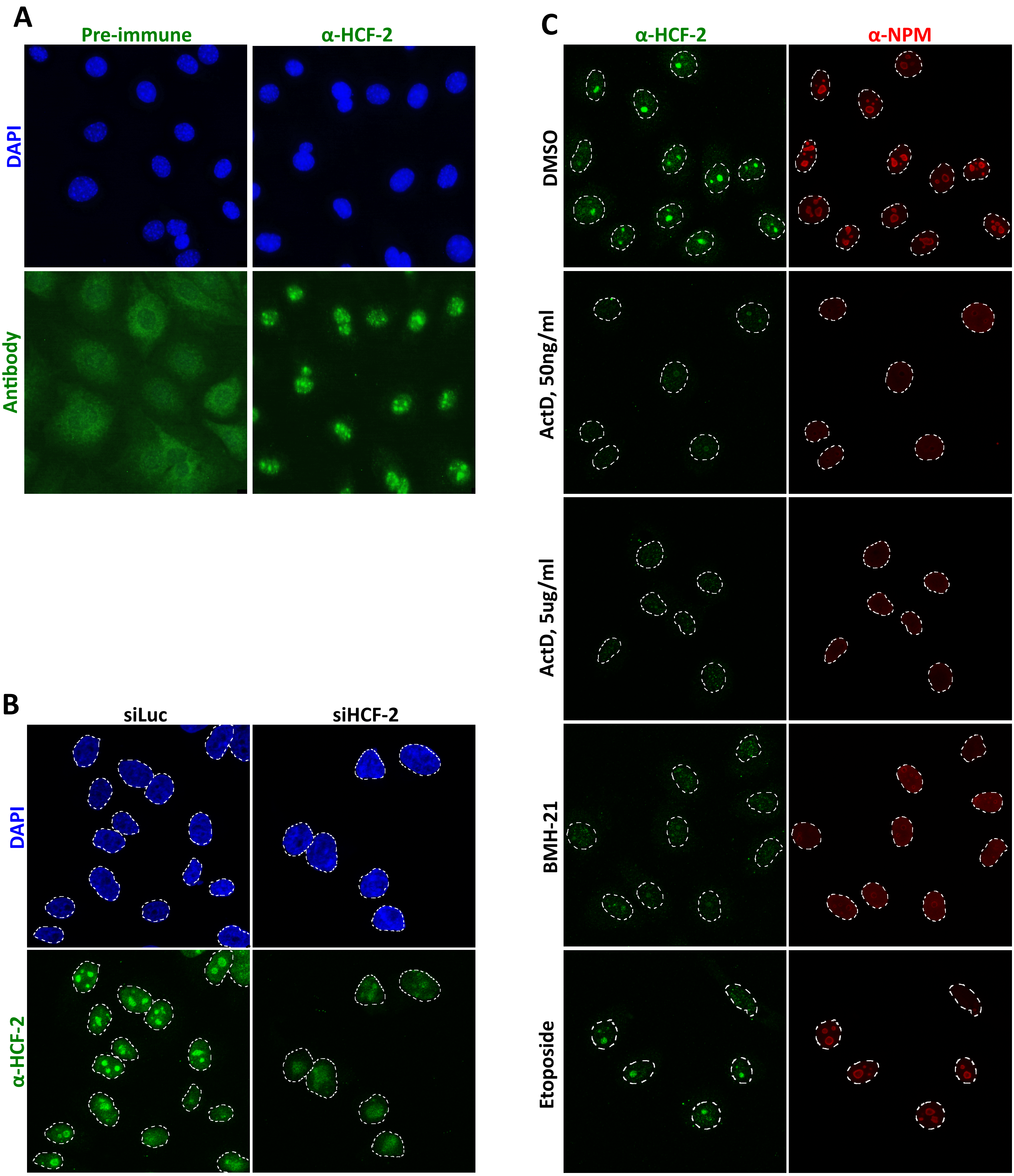
A
Pre-immune
α-HCF-2
DAPI
Antibody
B
siLuc
siHCF-2
DAPI
α-HCF-2
C
α-HCF-2
α-NPM
DMSO
ActD, 50ng/ml
ActD, 5ug/ml
BMH-21
Etoposide

Figure S3

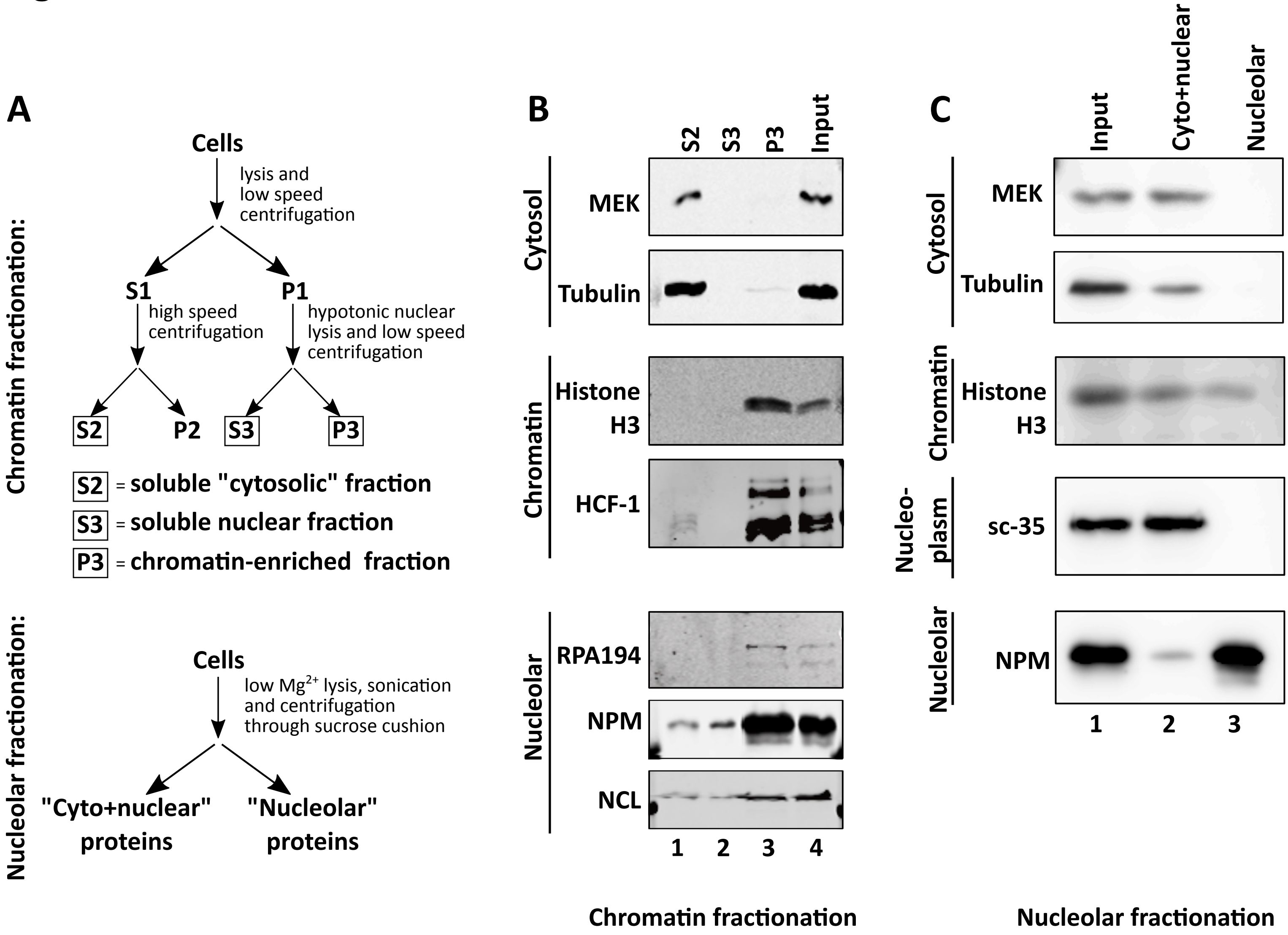
A
Chromatin fractionation:
Cells
lysis and
low speed
centrifugation
S1
P1
high speed
centrifugation
hypotonic nuclear
lysis and low speed
centrifugation
S2
P2
S3
P3
S2 = soluble "cytosolic" fraction
S3 = soluble nuclear fraction
P3 = chromatin-enriched fraction
Nucleolar fractionation:
Cells
low Mg2+ lysis, sonication
and centrifugation
through sucrose cushion
"Cyto+nuclear"
proteins
"Nucleolar"
proteins
B
S2
S3
P3
Input
Cytosol
MEK
Tubulin
Chromatin
Histone
H3
HCF-1
Nucleolar
RPA194
NPM
NCL
1
2
3
4
Chromatin fractionation
C
Input
Cyto+nuclear
Nucleolar
Cytosol
MEK
Tubulin
Chromatin
Histone
H3
Nucleo-
plasm
sc-35
Nucleolar
NPM
1
2
3
Nucleolar fractionation

Figure S4

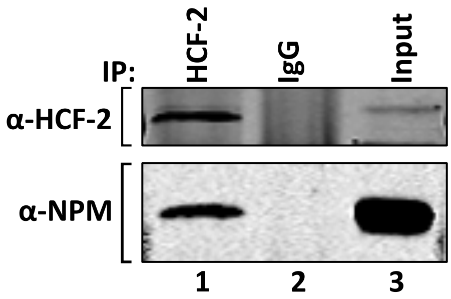

Figure S5

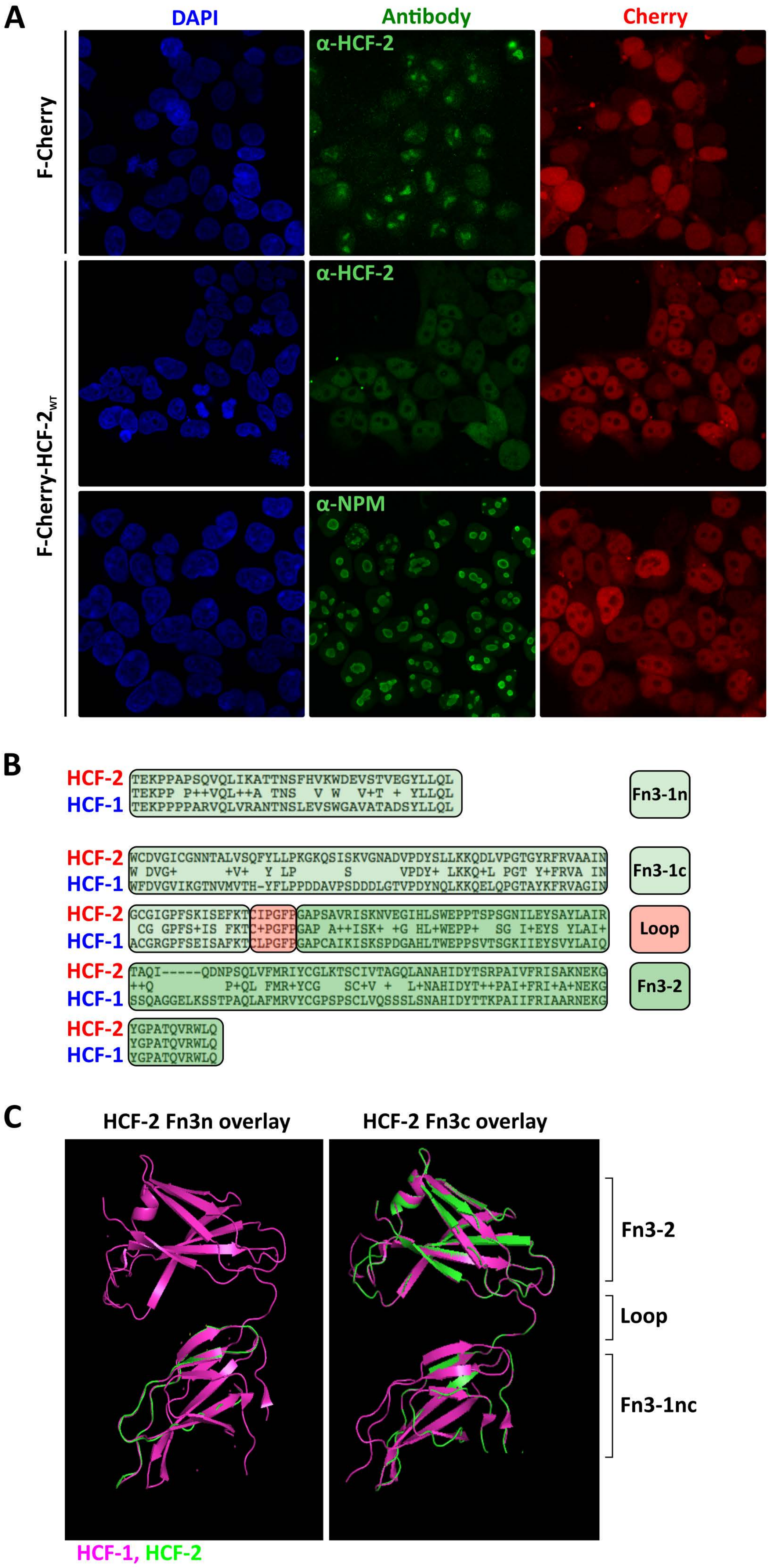
A
DAPI
Antibody
Cherry
F-Cherry
F-Cherry-HCF-2WT
α-HCF-2
α-HCF-2
α-NPM
B
HCF-2 TEKPPAPSQVQLIKATTNSFHVKWDEVSTVEGYLLQL
TEKPP P++VQL++A TNS V W V+T + YLLQL
HCF-1 TEKPPPPARVQLVRANTNSLEVSWGAVATADSYLLQL
Fn3-1n
HCF-2 WCDVGICGNNTALVSQFYLLPKGKQSISKVGNADVPDYSLLKKQDLVPGTGYRFRVAAIN
W DVG+ +V+ Y LP S VPDY+ LKKQ+L PGT Y+FRVA IN
HCF-1 WFDVGVIKGTNVMVTH-YFLPPDDAVPSDDDLGTVPDYNQLKKQELQPGTAYKFRVAGIN
Fn3-1c
HCF-2 GCGIGPFSKISEFKTCIPGFPGAPSAVRISKNVEGIHLSWEPPTSPSGNILEYSAYLAIR
CG GPFS+IS FKTC+PGFPGAP A++ISK+ +G HL+WEPP+ SG I+EYS YLAI+
HCF-1 ACGRGPFSEISAFKTCLPGFPGAPCAIKISKSPDGAHLTWEPPSVTSGKIIEYSVYLAIQ
Loop
HCF-2 TAQI-----QDNPSQLVFMRIYCGLKTSCIVTAGQLANAHIDYTSRPAIVFRISAKNEKG
++Q P+QL FMR+YCG SC+V + L+NAHIDYT++PAI+FRI+A+NEKG
HCF-1 SSQAGGELKSSTPAQLAFMRVYCGPSPSCLVQSSSLSNAHIDYTTKPAIIFRIAARNEKG
Fn3-2
HCF-2 YGPATQVRWLQ
YGPATQVRWLQ
HCF-1 YGPATQVRWLQ
C
HCF-2 Fn3n overlay
HCF-2 Fn3c overlay
Fn3-2
Loop
Fn3-1nc
HCF-1, HCF-2

Figure S6

A

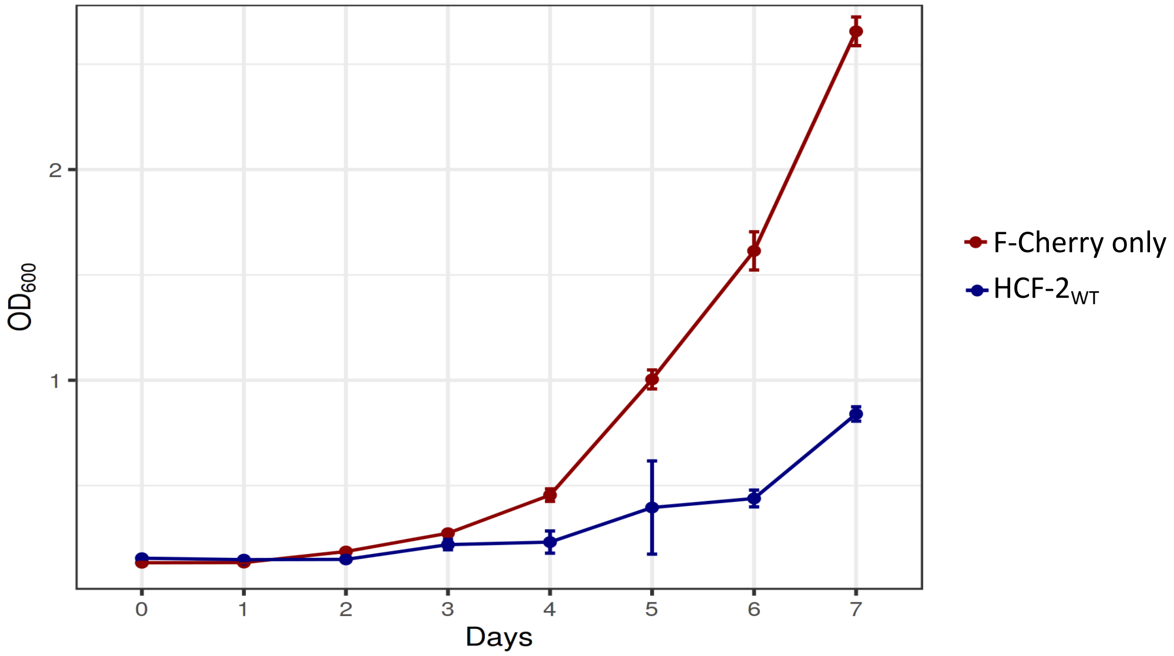

B

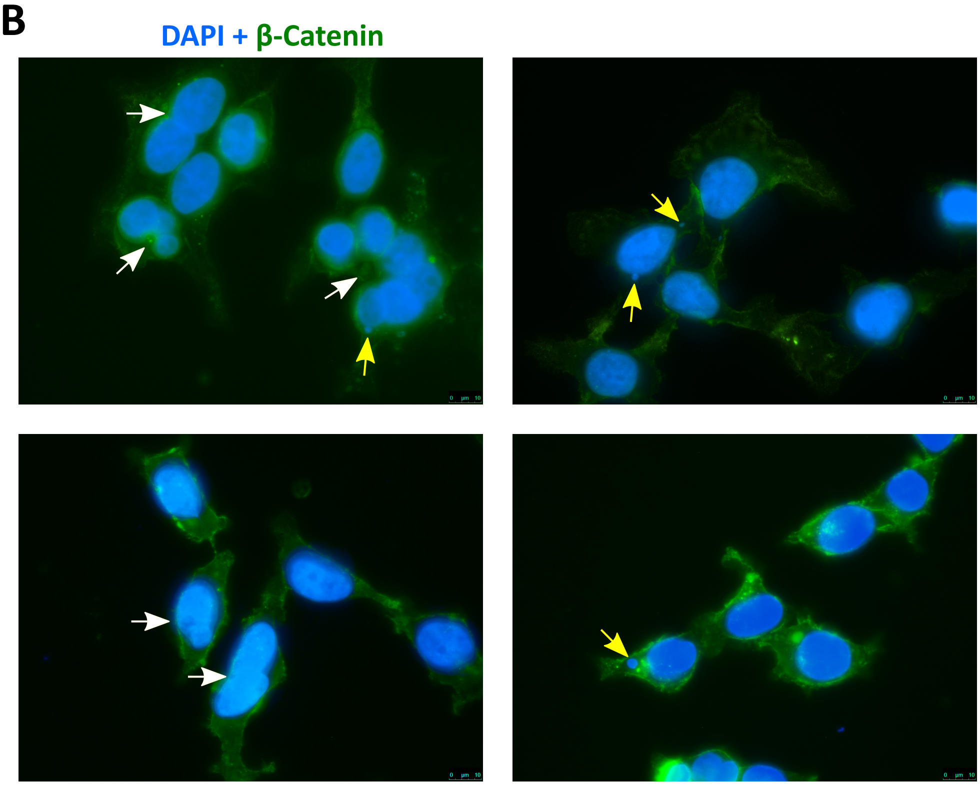

Figure S7

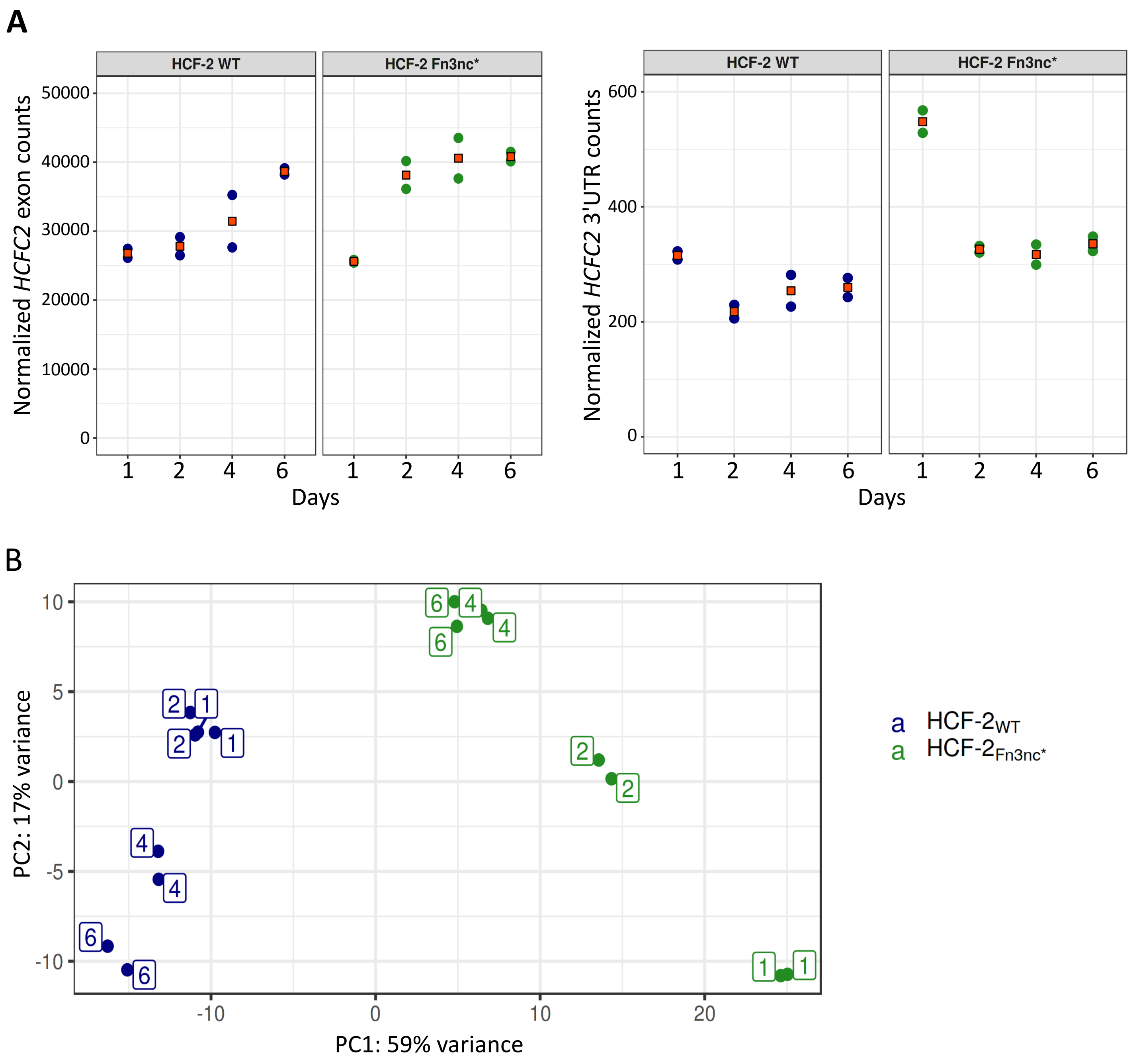
A
HCF-2 WT
HCF-2 Fn3nc*
Normalized HCFC2 exon counts
50000
40000
30000
20000
10000
0
1 2 4 6
Days
HCF-2 WT
HCF-2 Fn3nc*
Normalized HCFC2 3'UTR counts
600
400
200
0
Days
B
PC2: 17% variance
PC1: 59% variance
10
5
0
-5
-10
20
a HCF-2WT
a HCF-2Fn3nc*

Figure S8

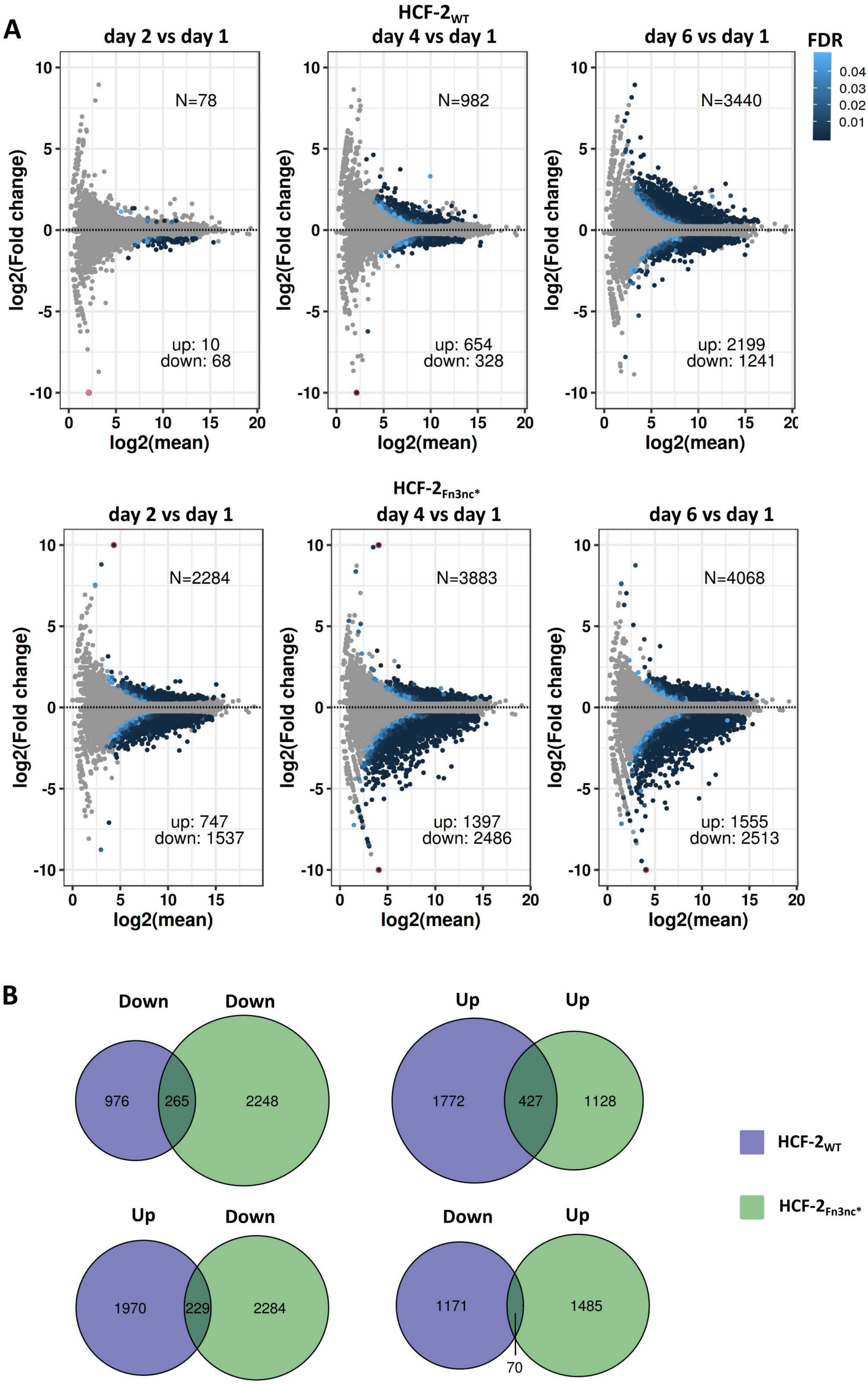

A
HCF-2WT
day 2 vs day 1
day 4 vs day 1
day 6 vs day 1
FDR
0.04
0.03
0.02
0.01
N=78
up: 10
down: 68
N=982
up: 654
down: 328
N=3440
up: 2199
down: 1241
log2(Fold change)
log2(mean)
HCF-2Fn3nc*
day 2 vs day 1
day 4 vs day 1
day 6 vs day 1
N=2284
up: 747
down: 1537
N=3883
up: 1397
down: 2486
N=4068
up: 1555
down: 2513
B
Down
Down
976
265
2248
Up
Up
1772
427
1128
Up
Down
1970
229
2284
Down
Up
1171
1485
70
HCF-2WT
HCF-2Fn3nc*

Figure S9

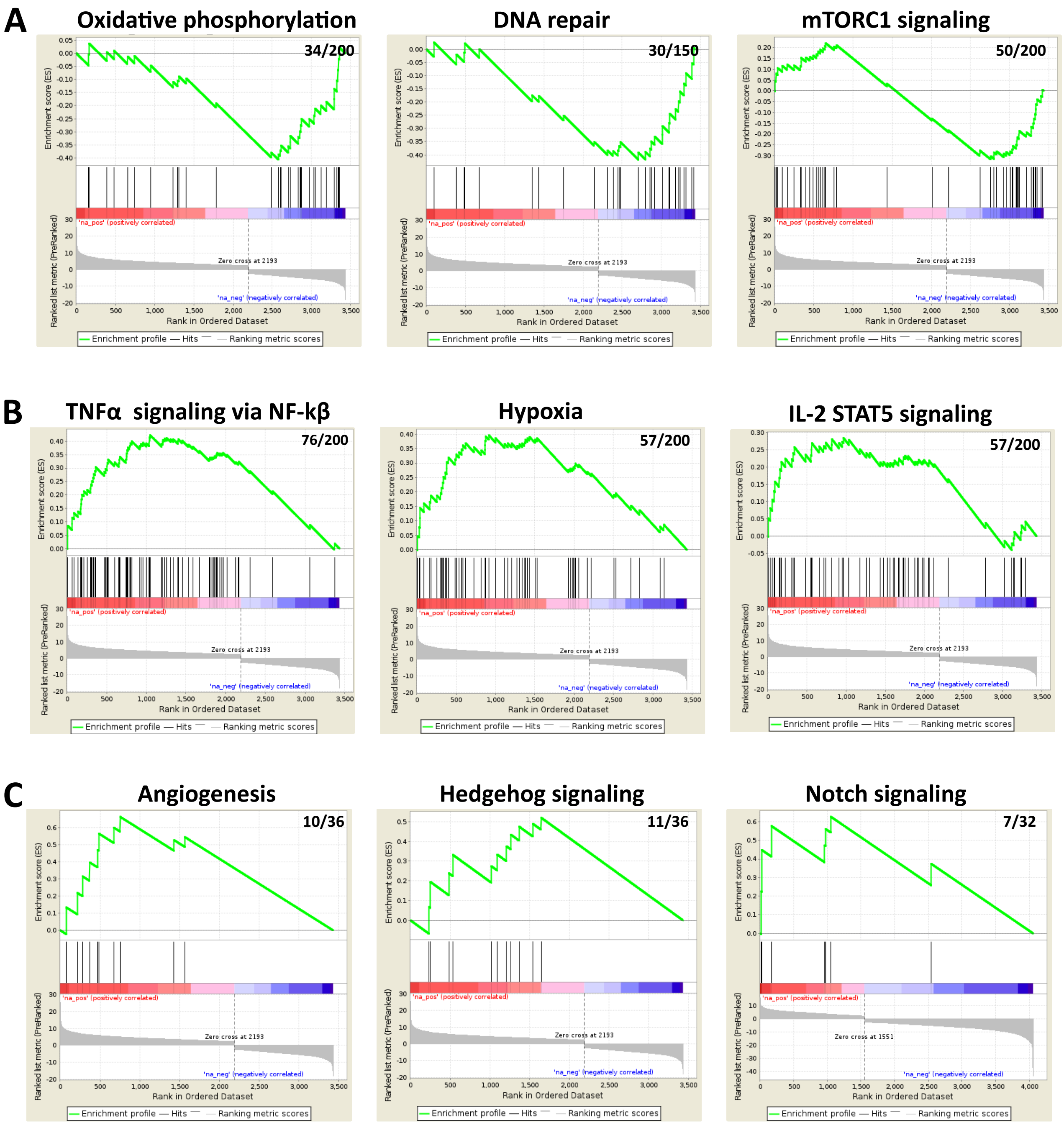
A
Oxidative phosphorylation
34/200
DNA repair
30/150
mTORC1 signaling
50/200
B
TNFα signaling via NF-kβ
76/200
Hypoxia
57/200
IL-2 STAT5 signaling
57/200
C
Angiogenesis
10/36
Hedgehog signaling
11/36
Notch signaling
7/32
Enrichment score (ES)
Ranked list metric (PreRanked)
'na_pos' (positively correlated)
'na_neg' (negatively correlated)
Zero cross at 2193
Zero cross at 1551
Rank in Ordered Dataset
Enrichment profile — Hits — Ranking metric scores

Figure S10

Cleavage (at position 10, E); Thr-rich (positions 14–21)

HCF-1$_{PRO}$ repeat		T	L	V	C	S	N	P	P	C	E	T	H	E	T	G	T	T	N	T	A	T	T	T	V	V	A
Chimaera	HCF-1 repeat #1	P	V	K	•	•	•	•	•	H	•	•	S	•	•	N	•	•	•	•	S	•	•	•	T	A	N
Chimaera	HCF-1 repeat #2	Q	G	I	P	E	S	V	H	G	•	•	A	T	A	•	•	•	•	•	•	•	•	S	S	T	•
Chimaera	HCF-2 repeat #1	A	Q	T	T	E	E	•	A	H	•	•	T	•	•	N	•	•	•	•	•	•	S	S	T	A	N
Chimaera	HCF-2 repeat #2	N	G	I	P	E	S	V	H	G	•	•	A	T	A	•	•	•	•	•	•	•	•	V	S	A	•

Figure S11

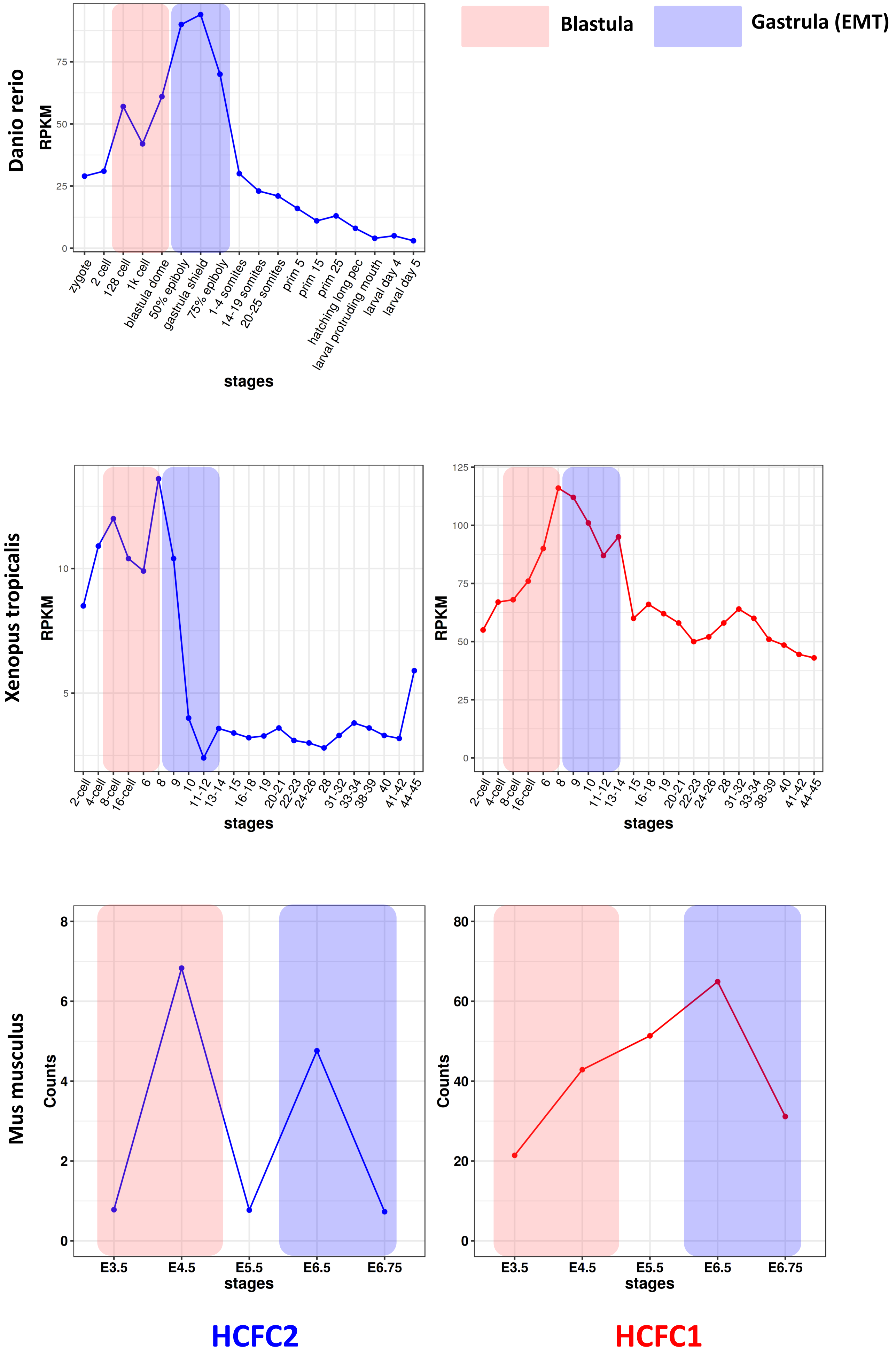
Blastula
Gastrula (EMT)
Danio rerio
RPKM
zygote
2 cell
128 cell
1k cell
blastula dome
50% epiboly
gastrula shield
75% epiboly
1-4 somites
14-19 somites
20-25 somites
prim 5
prim 15
prim 25
hatching long pec
larval protruding mouth
larval day 4
larval day 5
stages
Xenopus tropicalis
RPKM
2-cell
4-cell
8-cell
16-cell
6
8
9
10
11-12
13-14
15
16-18
19
20-21
22-23
24-26
28
31-32
33-34
38-39
40
41-42
44-45
stages
Mus musculus
Counts
E3.5
E4.5
E5.5
E6.5
E6.75
stages
HCFC2
HCFC1
